# Identification of crucial lncRNAs and mRNAs in liver regeneration after portal vein ligation through weighted gene correlation network analysis

**DOI:** 10.1186/s12864-022-08891-0

**Published:** 2022-09-21

**Authors:** Yan Zhu, Zhishuai Li, Jixiang Zhang, Mingqi Liu, Xiaoqing Jiang, Bin Li

**Affiliations:** 1 Department of Pathology, Changhai Hospital, Secondary Military Medicine University, Shanghai, 200433 China; 2 Biliary Tract Surgery Department I, Eastern Hepatobiliary Surgery Hospital, Secondary Military Medicine University, 225 Changhai Road, Yangpu, Shanghai, 200438 People’s Republic of China

**Keywords:** Portal vein occlusion, Liver regeneration, lncRNA, Transcriptome, WGCNA

## Abstract

**Background:**

Portal vein ligation (PVL)-induced liver hypertrophy increases future liver remnant (FLR) volume and improves resectability of large hepatic carcinoma. However, the molecular mechanism by which PVL facilitates liver hypertrophy remains poorly understood.

**Methods:**

To gain mechanistic insight, we established a rat PVL model and carried out a comprehensive transcriptome analyses of hepatic lobes preserving portal blood supply at 0, 1, 7, and 14-day after PVL. The differentially expressed (DE) long-non coding RNAs (lncRNAs) and mRNAs were applied to conduct weighted gene co-expression network analysis (WGCNA). LncRNA-mRNA co-expression network was constructed in the most significant module. The modules and genes associated with PVL-induced liver hypertrophy were assessed through quantitative real-time PCR.

**Results:**

A total of 4213 DElncRNAs and 6809 DEmRNAs probesets, identified by transcriptome analyses, were used to carry out WGCNA, by which 10 modules were generated. The largest and most significant module (marked in black_M6) was selected for further analysis. Gene Ontology (GO) analysis of the module exhibited several key biological processes associated with liver regeneration such as complement activation, IL-6 production, Wnt signaling pathway, autophagy, etc. Sixteen mRNAs (Notch1, Grb2, IL-4, Cops4, Stxbp1, Khdrbs2, Hdac2, Gnb3, Gng10, Tlr2, Sod1, Gosr2, Rbbp5, Map3k3, Golga2, and Rev3l) and ten lncRNAs (BC092620, AB190508, EF076772, BC088302, BC158675, BC100646, BC089934, L20987, BC091187, and M23890) were identified as hub genes in accordance with gene significance value, module membership value, protein–protein interaction (PPI) and lncRNA-mRNA co-expression network. Furthermore, the overexpression of 3 mRNAs (Notch1, Grb2 and IL-4) and 4 lncRNAs (BC089934, EF076772, BC092620, and BC088302) was validated in hypertrophic liver lobe tissues from PVL rats and patients undergoing hepatectomy after portal vein embolization (PVE).

**Conclusions:**

Microarray and WGCNA analysis revealed that the 3 mRNAs (Notch1, Grb2 and IL-4) and the 4 lncRNAs (BC089934, EF076772, BC092620 and BC088302) may be promising targets for accelerating liver regeneration before extensive hepatectomy.

**Supplementary Information:**

The online version contains supplementary material available at 10.1186/s12864-022-08891-0.

## Introduction

Hepatocellular carcinoma (HCC) is one of the common fatal malignant tumors with incidence rate ranking the sixth and cancer-related mortality rate ranking the third worldwide [1, 2]. HCC is the main subtype of primary liver cancer, and it accounts for 90% of this disease [3, 4]. Currently, curative hepatectomy is the first-line treatment choice for liver malignancies, and extended hepatectomy is required in the majority to achieve adequate surgical margins [5]. Although extensive hepatectomy is the best option for long-term survival in patients with large HCC, this treatment modality is commonly contraindicated because of insufficient future liver remnant (FLR) and consequent posthepatectomy liver failure (PHLF) [6, 7].

Mounting evidence has demonstrated that portal vein embolization or ligation (PVE/PVL) is the most acceptable method to achieve adequate FLR volume and decrease the risk of PHLF, thus enhances the resectability of initially unresectable large HCC [8–10]. PVE/PVL facilitates atrophy of the embolized liver lobe and concurrently induce compensatory hypertrophy of contralateral un-embolized liver. Increasing evidence from clinical trials has confirmed that PVE/PVL is correlated with a minimal mortality rate in patients with large HCC receiving extensive hepatectomy [7, 11–13]. Basic studies have revealed the partial mechanisms responsible for liver regeneration after PVE. Kawai et al., reported that PVE distends portal vein branches in non-embolized liver, and causing the exposure of liver blood vessels to stretch stress. Consequently, the hemodynamic change promotes the generation of Interleukin-6 (IL-6, a necessary early signal in liver regeneration) from endothelial cells and subsequent activation of regenerative cascades [14]. PVE causes a differential expression of transforming growth factor-alpha (TGF-α) and transforming growth factor-beta (TGF-β) between embolized lobes and non-embolized lobes, which might be associated with hepatocyte apoptosis and atrophy of the embolized lobes, and hepatocyte proliferation and hypertrophy of the un-embolized lobes [15]. However, the molecular mechanism by which PVE/PVL facilitates hypertrophy of hepatic lobes preserving portal blood supply (lobe-pbs) remains poorly understood.

Long noncoding RNA (lncRNA) is a class of functional RNA transcripts over 200 nucleotides in length. Although lncRNAs do not encode proteins, they exert key regulatory roles in various biological processes including development, cell differentiation, cell apoptosis, and cell proliferation [16, 17]. In fact lncRNAs are expressed at a more cell type- or tissue-specific manner than messenger RNAs (mRNA), indicating that lncRNAs possess distinct biological roles in physiological and pathological processes [18]. Although the importance of lncRNA has been widely demonstrated in liver regeneration following PVL or associated liver partition and portal vein ligation for staged hepatectomy (ALPPS) [17, 19–21], a comprehensive analysis of lncRNA-mRNA co-expression profiles in non-embolized lobe after PVL is lacking.

In the study, a rat PVL model was established and lobe-pbs tissues were collected at different time points (0, 1, 7, and 14 day) to carry out microarray analysis. Weighted gene correlation network analysis (WGCNA) was used to identify the cores of gene networks by calculating pairwise Pearson correlation matrix between all pairs of genes across all samples [22, 23]. A total of 10 modules were generated through WGCNA. Gene ontology (GO) analysis were next performed to reveal the key biological processes in these modules. The analysis of module-stage relationships was carried out to identify the most significant module. Finally, the hub mRNAs and lncRNAs associated with liver regeneration after PVL/PVE were identified and validated.

## Materials and methods

### Experimental design

**Fig. 1:**
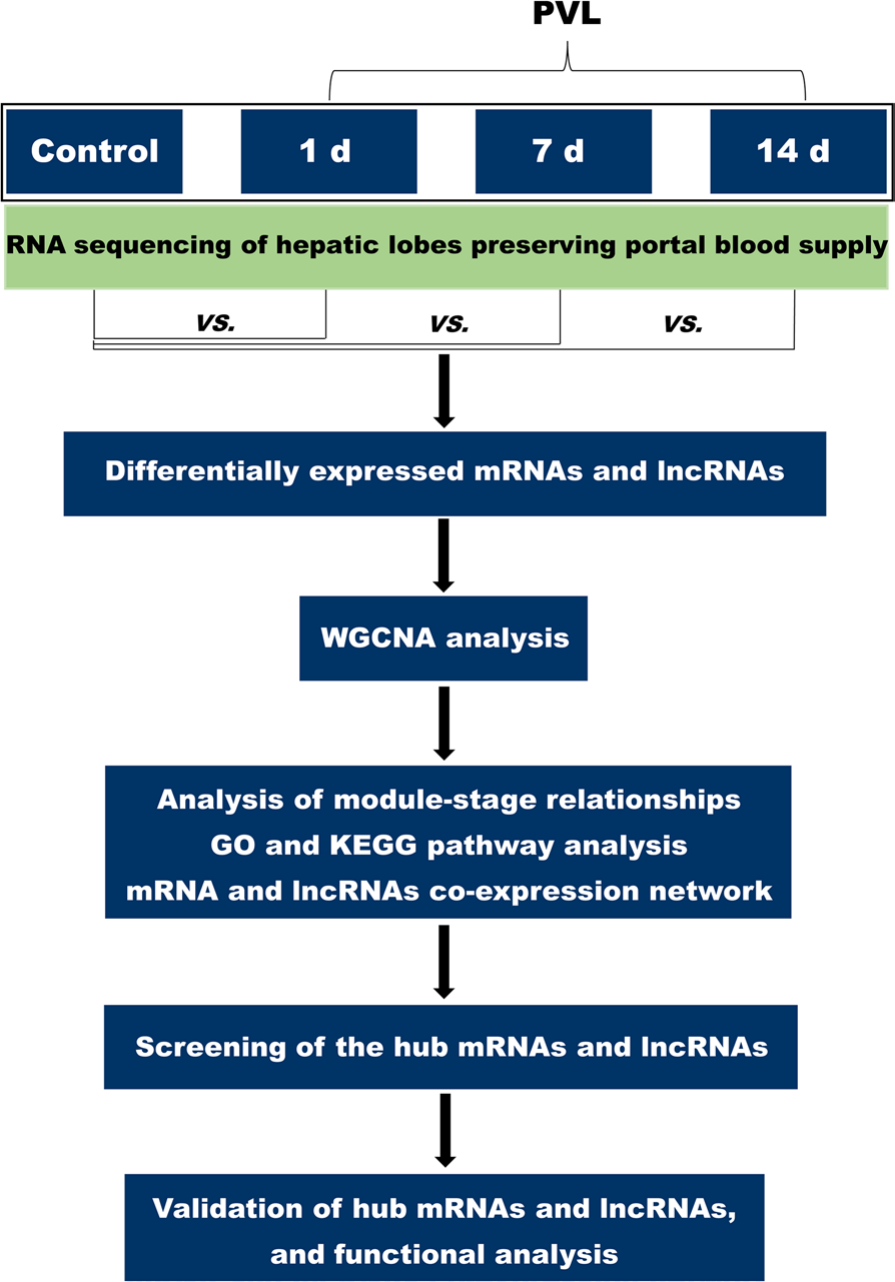
The overall workflow of this study

A schematic diagram depicting the detailed process in the present study was shown in Fig. 1.

### A rat PVL model

Six-eight weeks old male SD rats were purchased from the Animal Center of the Second Military Medical University (SMMU), and housed in optimal humidity (35–55%) and temperature (20–24 °C) with a 12 h light/12 h dark cycle and ad libitum access to water and food. The animal experiments were approved by the local Ethical Committee for Animal Experiments of the Eastern Hepatobiliary Surgery Hospital affiliated to SMMU (Grant number: DWLL-122). The study was carried out in compliance with the ARRIVE guidelines and in compliance with relevant guidelines and regulations such as the Animals (Scientific Procedures) Act 1986 in the UK and Directive 2010/63/EU in Europe.

A rat PVL model was constructed as described in our previous report [10]. In brief, the abdomen cavity was carefully opened under anesthesia with an intraperitoneal injection of pentobarbital-Na (40 mg/kg). Hepatic artery and bile duct were separated to keep unwounded. The left middle portal vein and hepatic papillary were separated from right portal vein, and double-ligated to obstruct blood supply. A rat PVL model was deemed successful when the right liver lobe remains light brown, and the other liver lobes turn dark brown. Rats were euthanized and whole livers were removed at different time points (0, 1, 7 and 14 d) after PVL. Euthanasia of rats is carried out with reference to the American Veterinary Medical Association (AVMA) Guidelines for Euthanasia of Animals (2020).

### Microarray analysis

Total RNA was extracted from lobe-pbs with Trizol Reagent (Thermo Fisher Scientific, MA, USA), and RNA quantity was evaluated using NanoDrop ND-1000 Spectrophotometer (Thermo Fisher Scientific).

The Agilent rat LncRNA Array v2.0 (4 × 44 K, Arraystar) was designed for profiling rat lncRNAs and mRNAs. A total of 10 333 lncRNAs and 28 287 mRNAs were assembled from comprehensive databases (RefSeq, Ensemble, Gencode, UCSC known genes, etc.). The microarray analysis was carried out at KangChen Bio-tech Corporation (Shanghai, Chain). In brief, RNA samples were used to hybridize to array slides after labeling. After washing, slides were scanned using an Agilent Microarray Scanner, and then data were collected with Agilent Feature Extraction software. An Agilent GeneSpring GX v11.5.1 software was used to normalize the collected data.

### Establishment of gene modules using WGCNA

The differentially expressed mRNAs (DEmRNAs) and differentially expressed lncRNAs (DElncRNAs) were first identified at four different time points (0, 1, 7, and 14 d) according to the following parameter: p-value < 0.05 and absolute fold change (FC) > 2. A total of 4213 DElncRNAs and 6809 DEmRNAs probesets were obtained from transcriptome analyses, and then the original probesets were converted to gene symbol. The gene co-expression network was constructed with WGCNA package in R software, as previously described [23–25]. In brief, the expression matrix was transformed into the adjacency matrix. Meantime β value (soft threshold power parameter) was calculated to assure a scale-free network. In the current study, soft threshold power β value was set as 18. Then the adjacency matrix was transformed into topological overlap matrix (TOM), and DElncRNAs and DEmRNAs were clustered in accordance with TOM-based dissimilarity measure. Hybrid tree cut was used to cut gene tree into 10 modules.

### Analysis of module-trait relationships

The relationships of co-expression modules with sample traits were evaluated in accordance with the phenotypic information at different time points. The association of co-expression modules with sample traits was calculated using Pearson’s correlation test, and *p*-value < 0.05 was considered significant. In the study, black_M6 module exhibited the highest correlation with sample traits and was selected for further analysis.

### Gene Ontology (GO) and Kyoto Encyclopedia of Genes and Genomes (KEGG) pathway analysis

GO functional and KEGG pathway enrichment analysis of DEmRNAs were carried out with DAVID v6.8 (the Database for Annotation, Visualization, and Integrated Discovery) [26, 27]. DAVID (http://david.abcc.ncifcrf.gov/) is an online bioinformatics tool offering the potential functional analysis of a number of mRNAs. GO categories with false discovery rate (FDR) < 0.05 were considered as significantly enriched, and KEGG pathways with a *p*-value < 0.05 were considered as significantly enriched. Only those GO categories or KEGG pathways contained ≥ 5 DEmRNAs were exhibited. GO was structured in three classes: biological process (BP), cellular component (CC), and molecular function (MF).

### Protein–Protein Interaction Network (PPI-network)

A total of 715 mRNAs were input into the STRING database (https://string-db.org/), a Search Tool for the Prediction of PPI-network, to construct the protein network [28]. Genes were sorted by betweeness decreasing, and the top 20 genes with larger betweeness were identified as key Genes.

### Identification of hub genes

The gene significance (GS) indicates the association of gene expression profile with sample trait, and the module membership (MM) indicates the association of gene expression profile with module eigengene. In the study the MM and GS values of each gene in the black_M6 module were calculated, and the correlation between MM and GS was analyzed before defining hub mRNAs and lncRNAs. The mRNAs (GS value > 0.5, MM value > 0.9) in the black_M6 module were used to construct PPI network. The top 20 genes with larger betweeness were identified as key mRNAs. The top 10 DElncRNAs with the smallest p-value of selected lncRNAs (GS > 0.5, MM > 0.9) from the M6 module were identified as key lncRNAs. These mRNAs and lncRNAs were used to construct lncRNA-mRNA co-expression network according to Pearson correlation coefficients. Cytoscape software 3.7.1 was applied to visualize lncRNA-mRNA co-expression networks. Finally, 16 hub mRNAs and 10 hub lncRNAs were identified.

### Quantitative real-time PCR (qRT-PCR)

Human liver tissues were obtained from EHBH. The study was conducted with the approval of the Ethics Committee of EHBH (Ethics Audit No. EHBHKY2020-K-004). Each patient signed the clinical study informed consent form in person or by proxy. The clinical study was conducted according to the principles expressed in the World Medical Association Declaration of Helsinki and in strict compliance with approved guidelines and regulations. Total RNA was extracted from twelve rat hyperplastic liver lobe after PVL and four (*n* = 4) patients underwent hepatectomy after PVE with Trizol Reagent (Thermo Fisher Scientific).

Reverse transcriptional PCR was carried out with Moloney’s murine leukemiavirus reverse transcriptase (Thermo Fisher Scientific) and random primers. qRT-PCR was carried out using SYBR green qPCR Master Mix (Thermo Fisher Scientific) on StepOnePlus Real-Time PCR System (Thermo Fisher Scientific). Beta-actin was used as the reference gene to normalize lncRNA and mRNA expression. Relative expression level of lncRNA and mRNA was counted through using 2^(−ΔΔCT)^ method.

### Statistical analysis

Data were present as the mean ± standard deviation. Statistical analysis was conducted using SPSS software v18.0 (IBM, NY, USA). The difference was compared using two-tailed student’s t-test, or one-way analysis of variance (ANOVA) followed by the Scheffé test. The difference was deemed significant at *p*-value < 0.05.

## Results

### DEmRNAs and DElncRNAs profiles in lobe-pbs after PVL

**Fig. 2:**
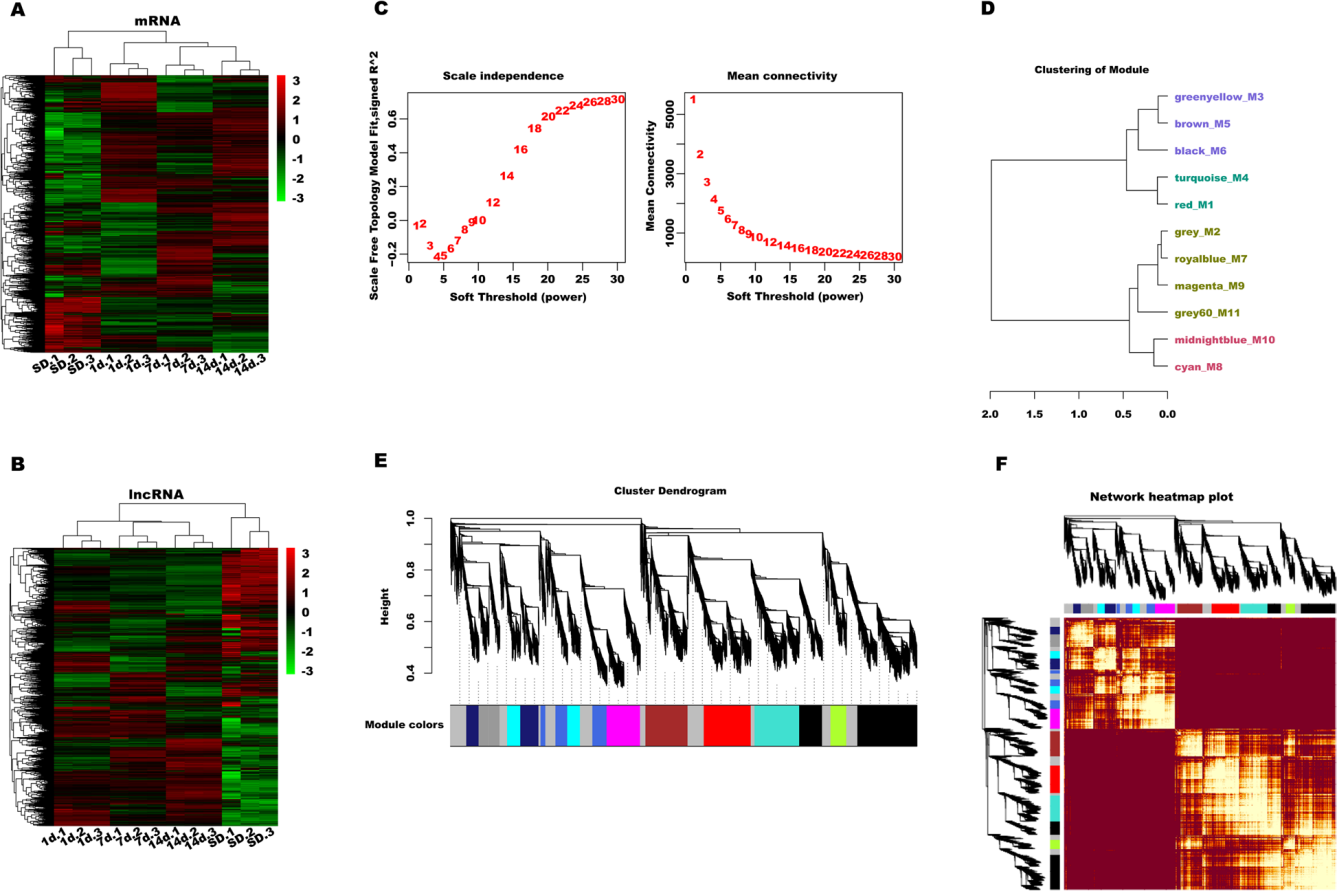
WGCNA based on microarray data. Hierarchical clustering analysis of mRNAs (**A**) and lncRNAs (**B**) differentially expressed in rat lobe-pbs at different time points (0, 1, 7 and 14 d) after PVL. (**C**) The analysis of network topology using different soft-thresholding power. (**D**) The cluster dendrogram in accordance with module eigengenes. (**E**) Cluster dendrogram displaying co-expression modules identified by WGCNA. LncRNAs and mRNAs that have highly similar expression pattern were clustered into the same module. (**F**) An adjacency heatmap revealed the topological overlap matrix among 4213 DElncRNAs and 6809 DEmRNAs probesets

To explore the underlying mechanism of lncRNAs on liver regeneration after PVL, the expression profiles of lncRNAs and mRNAs in lobe-pbs were analyzed at 0, 1, 7, and 14 days after PVL. Hierarchical cluster analysis revealed an extensive expression changes of lncRNAs and mRNAs in lobe-pbs at different time points (Fig. 2A and B). The results from microarray analysis showed that there were 3686 DEmRNAs and 2485 DElncRNAs probesets between control group and PVL day 1 (p-value < 0.05 and FC ≥ 2.0, similarly hereinafter), 2965 DEmRNAs and 2391 DElncRNAs probesets between control group and PVL day 7, and 3570 DEmRNAs and 2694 DElncRNAs probesets between control group and PVL day 14 (Supplementary fig. S1A and B). These differential probesets from three comparisons were united and 4213 DElncRNAs and 6809 DEmRNAs probesets were identified.

### WGCNA and key module identification

A total of 4213 lncRNAs and 6809 mRNAs were applied to conduct co-expression network analysis using WGCNA R software package. It is the most important to determine soft-thresholding power to the relative equalization between scale independence and mean connectivity [29]. In the study β = 18 was used to construct a hierarchical clustering tree after analyzing the network topology with soft-thresholding power from 1 to 30 (Fig. 2C). The lncRNAs and mRNAs with highly similar expression mode were put into a module using the dynamic hybrid tree cutting algorithm, and each module was designated a unique color. The analysis generated ten modules (black_M6, red_M1, turquoise_M4, brown_M5, magenta_M9, midnightblue_M10, royalblue_M7, cyan_M8, grey_M11, and greenyellow_M3) (Fig. 2D), an eigengene dendrogram (Fig. 2E), and adjacency heatmap (Fig. 2F). The number of genes in the 10 modules was showed in Table 1.

**Table 1:** The amount of mRNAs and lncRNAs in the 10 modules

Module	lncRNAs	mRNAs	All numbers
black_M6	648	1256	1904
red_M1	308	781	1089
turquoise_M4	328	712	1040
brown_M5	393	586	979
magenta_M9	406	369	775
midnightblue_M10	220	488	708
royalblue_M7	403	317	720
cyan_M8	229	369	598
grey_M11	194	286	480
greenyellow_M3	124	242	366

**Fig. 3:**
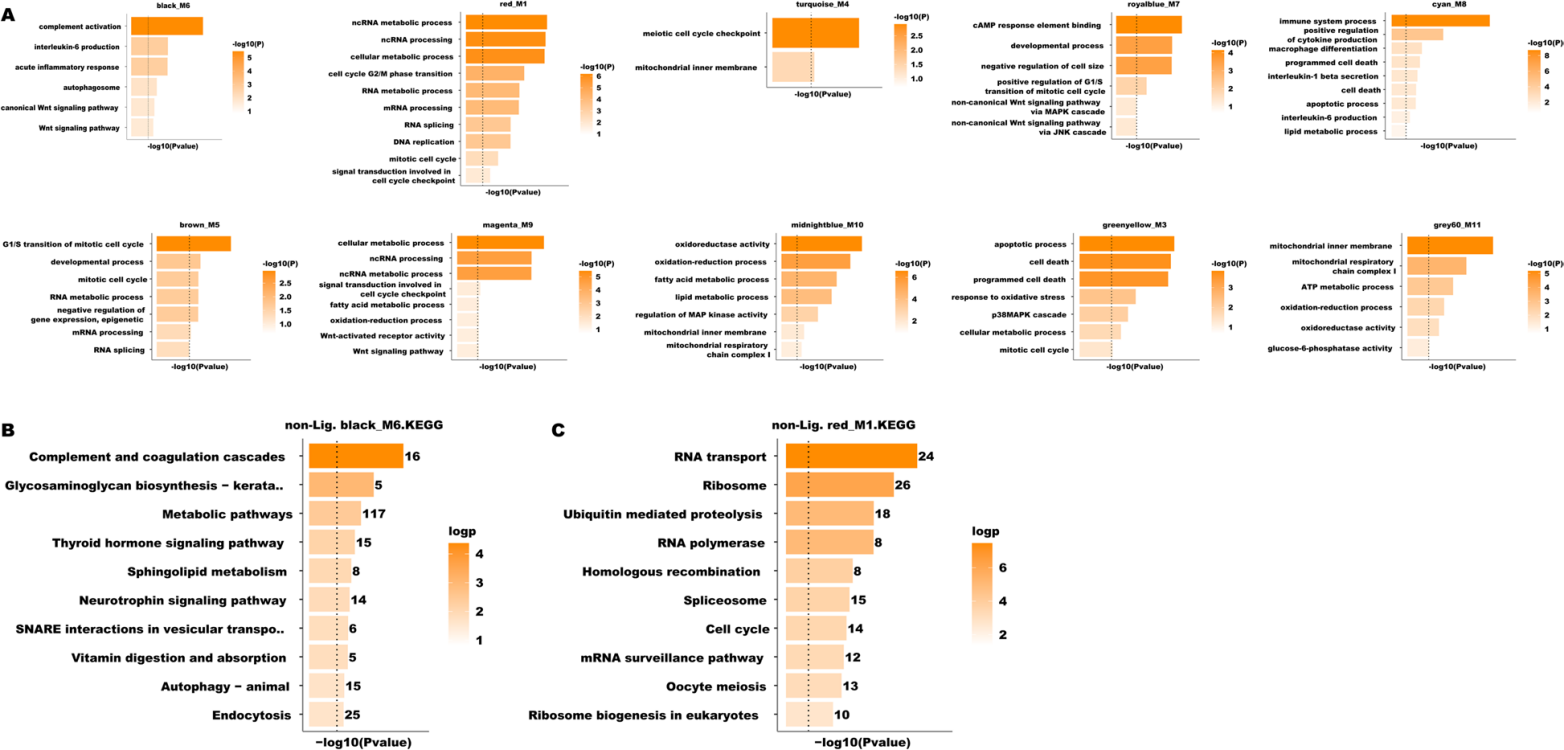
Functional enrichment analysis. (**A**) Representative GO terms in the 10 modules. (**B**) KEGG analysis of mRNAs showed in black_M6 module. (**C**) KEGG enrichment analysis of mRNAs showed in red_M1 module

Among the 10 modules, black_M6 possessed the highest number of DElncRNAs and DEmRNAs compared with other modules (Table 1). Moreover, GO analysis of the 10 modules showed some critical biological processes involved in liver regeneration (Fig. 3A). GO terms of black_M6 module showed that several genes involved in complement activation (*Mbl1*, *Crp*, *Cd46*, *Cft*, *Masp1*, *C1r*, etc.), IL-6 production (*Cd24*, *Aqp4*, *Sirpa Adora2b*, *Gas6*, *Nod2*, *and Myd88,* etc.), classical Wnt signaling pathway (*Fgfr2*, *Gpc3*, *Notch1*, *Fzd6*, *and Hdac2*, etc.), autophagy (*Map1lc3B*, *Vcp*, *Nod2*, *Atg12*, *Vps13d*, *and Rab12*, etc.), and acute inflammatory response (*C4pbpa*, *Ptger3*, *Cd46*, *Masp1*, *and Serping1*, etc.) were immediately increased after PVL and remained high up to day 14 post-PVL. Consistent with previous studies, these data indicated that complement activation [30], IL-6 production [31, 32], Wnt signaling pathway [33], and autophagy [34], were closely correlated with liver regeneration after PVL. KEGG enrichment analysis showed that complement and coagulation cascades, metabolic pathway, hippo signaling pathway, and autophagy were the most significant pathways in the module (Fig. 3B). Red_M1 module possessed the second highest number of DElncRNAs and DEmRNAs compared with other modules. GO terms of red_M1 module showed that several genes involved in non-coding (nc) RNA processing (*Rps7*, *Elp1*, *Rpf2*, *Trmt10a*, *and Ints11*, etc.), ncRNA metabolic process (*Tp53*, *Rpl14*, *Rps6*, *and Polr1b*, etc.), and RNA splicing (*Zranb2*, *Smn1*, *Hnrnpm*, *Hnrnpk*, *Hnrnpc*, *and Srsf10*, etc.) were immediately increased after PVL but then quickly reversed at day 7 post-PVL, and continued to slightly increase at day 14 post-PVL. KEGG enrichment analysis showed that RNA polymerase, ribosome, and cell cycle were the most significant pathways in the module (Fig. 3C). These data indicated that dys-regulated ncRNAs (lncRNAs, miRNAs, etc.) were closely correlated with liver regeneration after PVL. The significance of several biological processes remains to be elucidated.

### Analysis of module-stage relationships

**Fig. 4:**
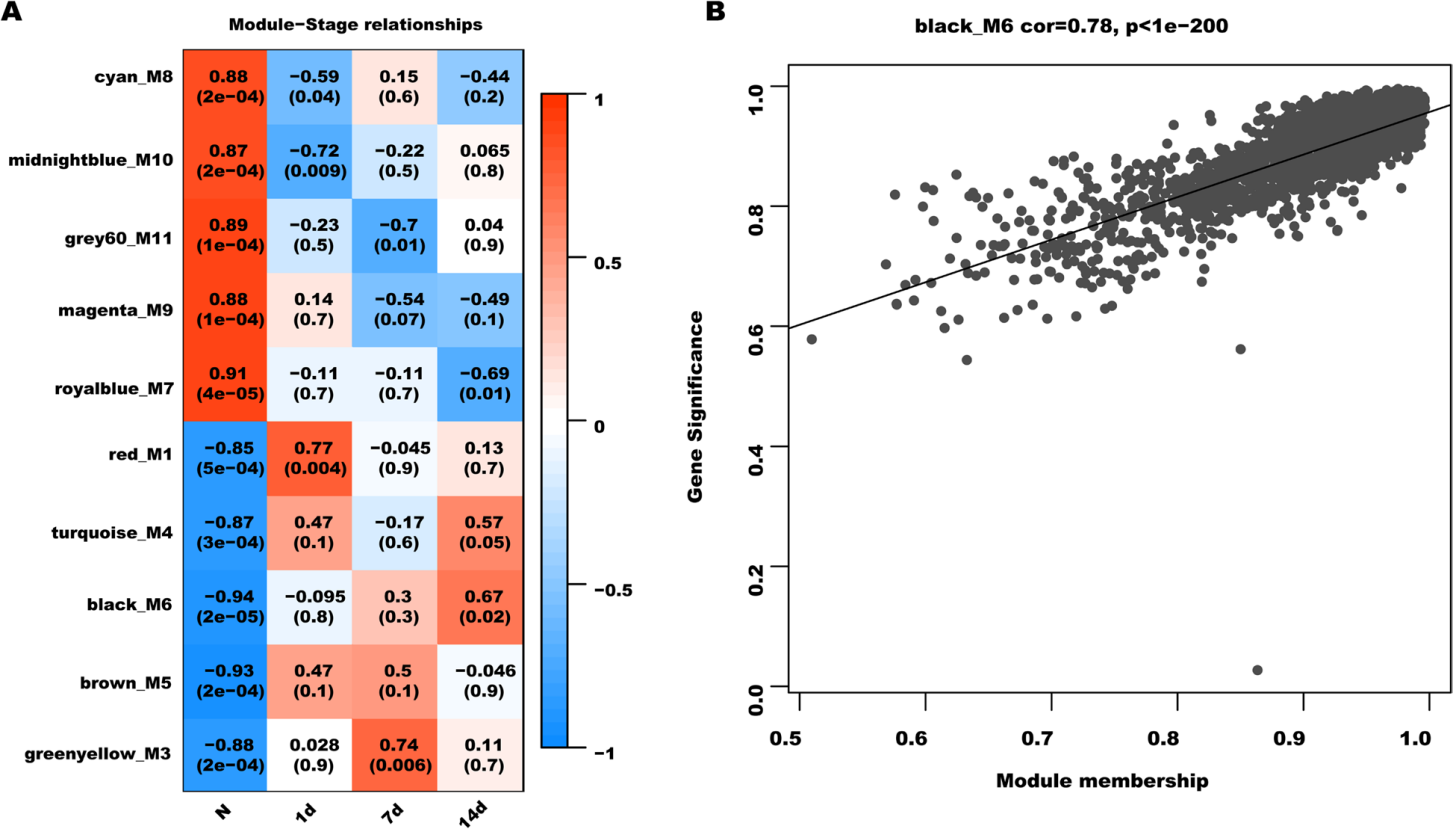
The analysis of module-stage relationships. (**A**) Module-stage relationships analysis. Every line represents a module eigengene. Every column represents a stage (time point). Every cell contains the correlation coefficient and p value. (**B**) A scatterplot of GS *vs.* MM in black_M6 module

The correlation between co-expression modules and particular traits was next identified. To this end, the r and p-value were set in module-stage correlation analysis. As shown in Fig. 4A, M6 module showed the highest correlation with liver regeneration after PVL (r = -0.94, *p*-value = 2 × 10^–5^) in these modules. M6 module also possessed the highest number of DElncRNAs and DEmRNAs compared with other modules (Table 1). Therefore, M6 module was selected for further analysis. Module membership (MM) illustrates intra-modular connectivity and gene significance (GS) illustrates the correlation of gene with time stage [35]. The scatter diagram analysis showed that GS value was prominently associated with MM value in the M6 module (r = 0.78, *p*-value = 1e-200, Fig. 4B). Therefore, the MM and GS values of genes in M6 module was calculated to identify hub lncRNAs and mRNAs. Through filtering with GS > 0.5 and MM > 0.9, 373 lncRNAs and 715 mRNAs in the M6 module were identified as key genes in liver regeneration (Supporting Table S1).

### Identification of hub mRNAs and lncRNAs

**Fig. 5:**
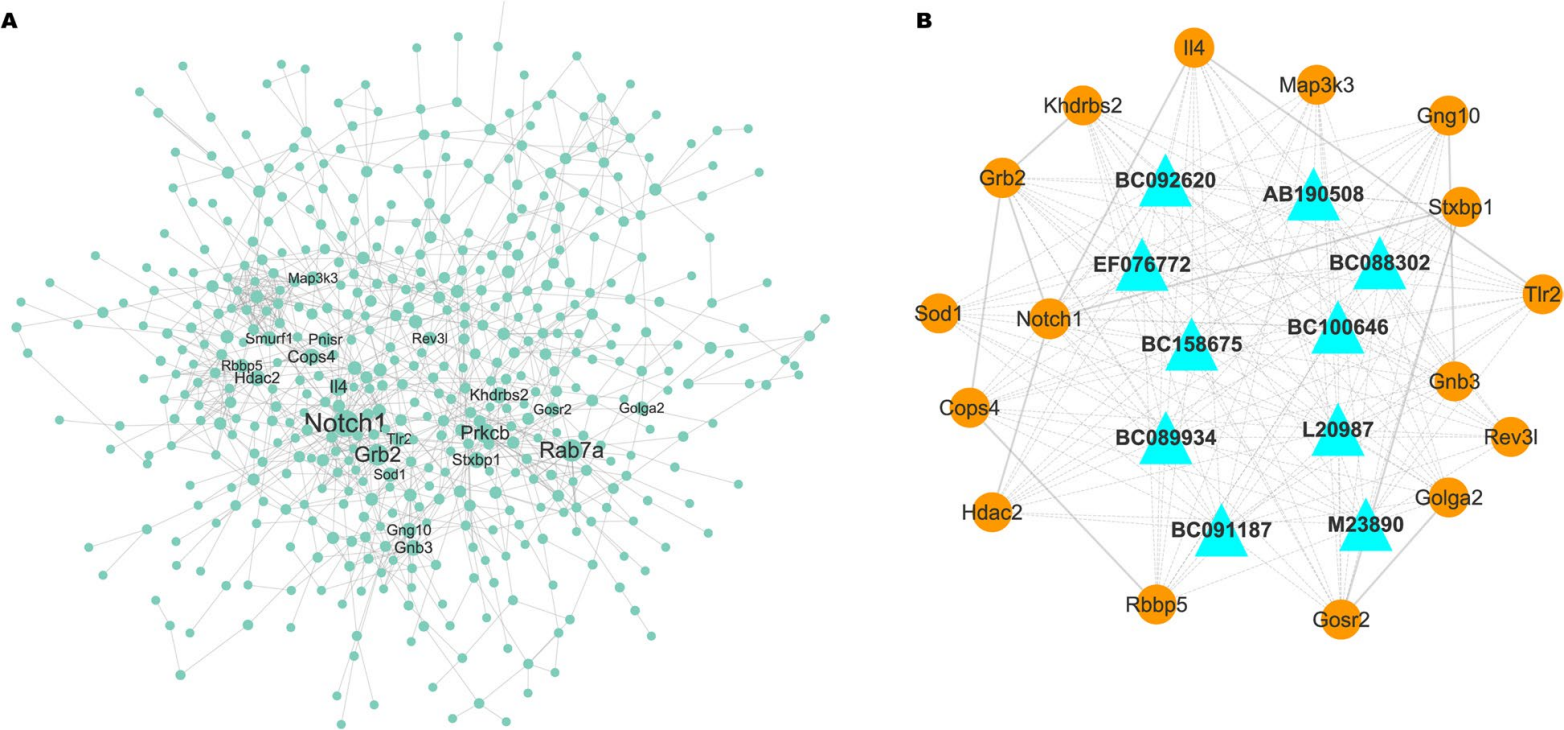
Identification of hub mRNAs and lncRNAs. (**A**) A key gene-associated PPI network with 488 nodes and 992 edges using STRING database and Cytoscape software based on betweeness centrality, and genes were sorted by betweeness decreasing. (**B**) A lncRNA-mRNA co-expression network was constructed using the key lncRNAs and mRNAs to identify 16 hub mRNAs (*Notch1*, *Grb2*, *IL-4*, *Cops4*, *Stxbp1*, *Khdrbs2*, *Hdac2*, *Gnb3*, *Gng10*, *Tlr2*, *Sod1*, *Gosr2*, *Rbbp5*, *Map3k3*, *Golga2*, and *Rev3l*) and 10 hub lncRNAs

We next constructed a key genes-associated PPI network with 488 nodes and 992 edges using STRING database and Cytoscape software based on betweeness centrality [36] and genes were sorted by betweeness decreasing (Fig. 5A). The top 20 genes with larger betweeness were identified as key mRNAs (*Notch1*, *Rab7a*, *Grb2*, *Prkcb*, *IL-4*, *Cops4*, *Stxbp1*, *Khdrbs2*, *Hdac2*, *Gnb3*, *Pnisr*, *Gng10*, *Tlr2*, *Smurf1*, *Sod1*, *Gosr2*, *Rbbp5*, *Map3k3*, *Golga2*, and *Rev3l*, Supporting Table S2). The top 10 DElncRNAs with the smallest p-value of selected lncRNAs (GS > 0.5, MM > 0.9) from the M6 module were identified as key lncRNAs (*BC092620*, *AB190508*, *EF076772*, *BC088302*, *BC158675*, *BC100646*, *BC089934*, *L20987*, *BC091187*, and *M23890*, Supporting Table S3). Hierarchical cluster analysis showed the expression variations of these hub lncRNAs and mRNAs in lobe-pbs at different time points (Supplementary figure S2A and B).

### Validation and functional analysis of hub mRNAs and lncRNAs

**Fig. 6:**
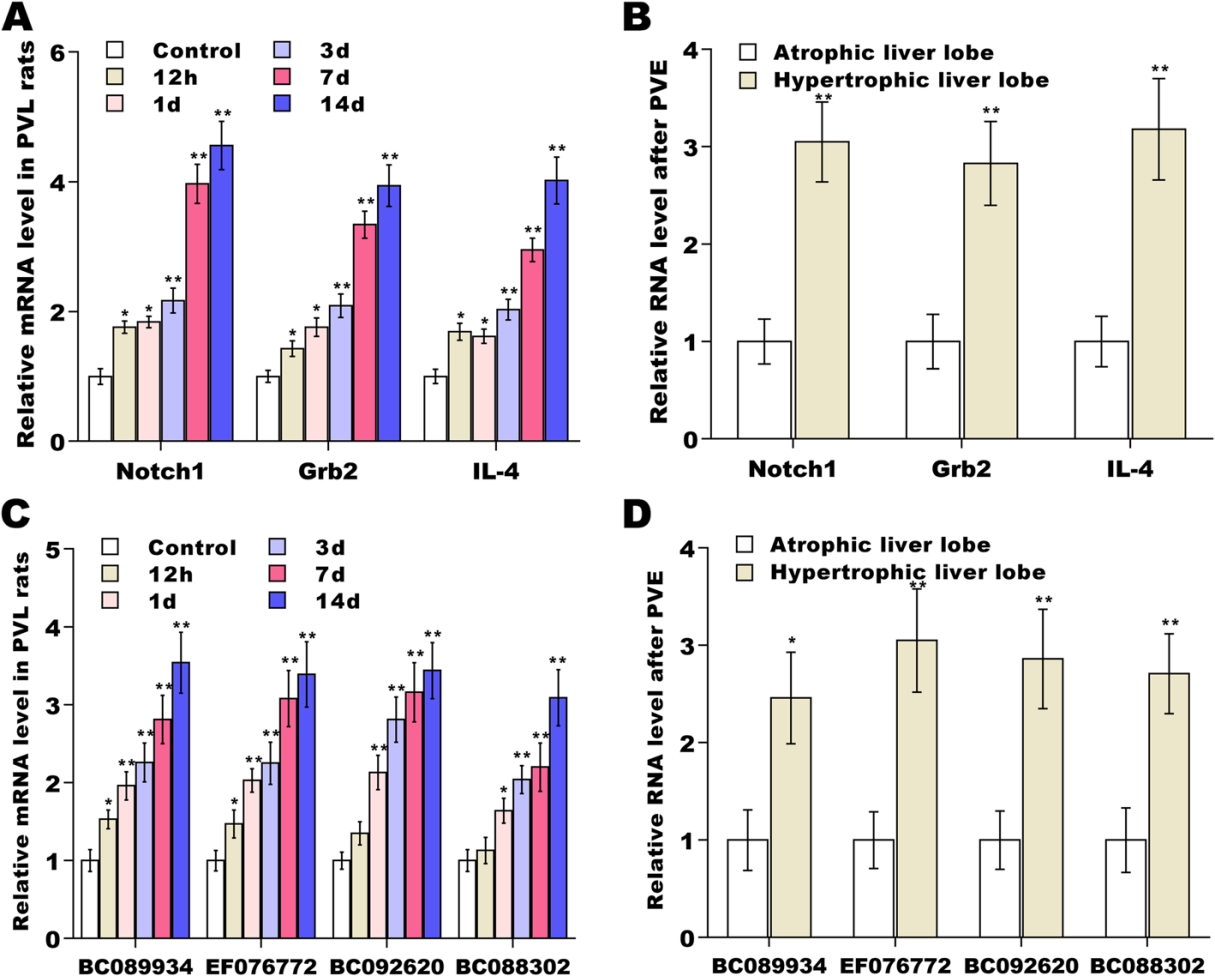
qRT-PCR analysis. (**A** and **C**) qRT-PCR analysis of 3 mRNAs (*Notch1*, *Grb2* and *IL-4*) and 4 lncRNAs (*BC089934*, *EF076772*, *BC092620*, and *BC088302*) in rat lobe-pbs at different time points (0, 0.5, 1, 3, 7 and 14 d) after PVL (*n* = 5). (**B** and **D**) qRT-PCR analysis of 3 mRNAs (*Notch1*, *Grb2* and *IL-4*) and 4 lncRNAs (*BC089934*, *EF076772*, *BC092620*, and *BC088302*) in hyperplastic and atrophic liver lobes in patients who underwent hepatectomy 3–4 weeks after the implementation of PVE (*n* = 4)

Then, we constructed a lncRNA-mRNA co-expression network using these key lncRNAs and mRNAs to identify 16 hub mRNAs (*Notch1*, *Grb2*, *IL-4*, *Cops4*, *Stxbp1*, *Khdrbs2*, *Hdac2*, *Gnb3*, *Gng10*, *Tlr2*, *Sod1*, *Gosr2*, *Rbbp5*, *Map3k3*, *Golga2*, and *Rev3l*) and 10 hub lncRNAs (Fig. 5B). LncRNAs were showed as triangle nodes and mRNAs were showed as circular nodes. Grey solid lines represented mRNA-mRNA interaction and grey dotted lines represented mRNA-lncRNA interaction. Several mRNAs (*Notch1*, *Grb2* and *IL-4*), co-expressed with multiple lncRNAs and mRNAs, were selected for further validation. As shown in Fig. 6A, the expression of *Notch1*, *Grb2* and *IL-4* was immediately increased after PVL and remained high up to day 14 post-PVL in rat model of PVL. The expression of *Notch1*, *Grb2* and *IL-4* was significantly increased in hyperplastic liver lobes in clinical samples undergoing hepatectomy 3–4 weeks after PVE (Fig. 6B). The expression of several lncRNAs (*BC089934*, *EF076772*, *BC092620*, and *BC088302*) was also validated, and Fig. 6C and D showed that the level of *EF076772*, *M23890*, *L20987*, and *BC100646* was increased in hyperplastic liver lobes tissues from PVL rats and patients undergoing hepatectomy after PVE.

The expression of *Notch1*, *Grb2* and *IL-4* was significantly increased in hypertrophic liver lobes compared with atrophic liver lobes after PVE (Fig. 6B). The expression of several lncRNAs (*BC089934*, *EF076772*, *BC092620*, and *BC088302*) was also validated, and Fig. 6C and D showed that the level of *EF076772*, *M23890*, *L20987*, and *BC100646* was increased in lobe-pbs tissues from PVL rats and in hypertrophic liver lobes from patients undergoing hepatectomy compared with atrophic liver lobes after PVE.

## Discussion

Hepatectomy is one of the best choice for curative treatment of patients with liver cancer [37]. Although the clinical outcome of hepatectomy has a marked improvement over the last few decades, numerous liver cancer patients are diagnosed at an unresectable stage (locally advanced or metastatic disease) and lost the opportunity to surgical treatment due to a small FLR and function [38, 39]. To enhance FLR and resectability of large HCC, many techniques have been developed, such as PVE, ALPPS, and radiation lobectomy[39]. All three treatments accelerate liver hypertrophy in liver cancer patients, but PVE is considered as the best choice based on the effective hypertrophy with a short time and low rate of complication [39]. In the study, our main aim was to uncover the crucial lncRNAs and mRNAs of PVL that promote liver regeneration similar to the mechanism of PVE.

Over the past few decades, a number of molecules and intracellular signaling pathways involved in liver regeneration have been identified. For instance, *IL-6* is an indispensable element in liver regeneration, and *IL-6* deletion impairs liver cell proliferation characterized by hepatic failure in *IL-6*-depleted mice [31, 40]. Furthermore, preoperative supplementation of *IL-6* restores hepatocyte proliferation and liver regeneration in *IL-6*-depleted mice [31]. Emerging studies have demonstrated that IL-6 contributes to activate a couple of signaling pathways involved in liver regeneration, such as *Jak/Stat3* pathway [41, 42], mitogen-activated protein kinase (*MAPK*) pathway [43], and *ERK* and *PKB* signaling [40]. In the current study, the results from GO analysis revealed that IL-6 production (*Cd24, Aqp4, Sirpa Adora2b, Gas6, Nod2, and Myd88,* etc.), together with classical *Wnt* signaling pathway (*Fgfr2*, *Gpc3*, *Notch1*, *Fzd6*, *and Hdac2*, etc.) and acute inflammatory response (*C4pbpa*, *Ptger3*, *Cd46*, *Masp1*, *and Serping1*, etc.) were immediately upregulated after PVL and remain high up to day 14 post-PVL. These data suggest that IL-6 is essential for liver regeneration, and that the present analysis is convincing.

The rapid advancement of microarray and next-generation sequencing techniques has highly expanded the ability to systemically analyze the molecular alterations during liver regeneration. To identify new immediate-early genes, Togo et al., conducted a cDNA microarray analysis at 1 and 3 h after hepatectomy, and cluster analysis showed that interleukin-1 receptor associated kinase-1, Karyopherin α1, inhibitor of DNA binding 2 and 3, and growth arrest and DNA-damage-inducible protein are closely correlated with early liver regeneration [44]. Recent evidence has demonstrated that lncRNAs play important roles in liver regeneration [20]. Xu et al., revealed that the levels of 1,231 lncRNAs and 3,141 mRNAs are dysregulated, and that upregulated lncRNA-LALR1 after 2/3 partial hepatectomy (PH) promotes hepatocyte proliferation in vitro and hepatic regeneration in vivo through activating Wnt signaling pathway [19]. Through microarray analysis, Huang et al., identified that 400 lncRNAs were differentially expressed after 2/3 PH [20]. Functionally, upregulated *lncPHx2* inhibits liver regeneration, whereas *lncPHx2* silencing results in a significantly increased hepatocyte proliferation [20].

Although plenty of functional lncRNAs and mRNAs have been identified during liver regeneration, the critical lncRNAs and mRNAs in liver regeneration after PVE/PVL remain unknown. PVE/PVL is carried out to accelerate compensatory hypertrophy of lobe-pbs. Given the role of PVE/PVL in increasing the resectability of initially unresectable large HCC, revealing the mechanism underlying PVE/PVL-induced liver regeneration is indispensable. We first carried out a microarray analysis to identify DElncRNAs and DEmRNAs in rats after PVL. A powerful bioinformatics algorithm, WGCNA, was next applied to analyze the relationship among DElncRNAs and DEmRNAs. Through WGCNA, the similar transcripts are classified into same modules and all modules are linked to clinical traits [29, 45]. In the study, a total of 4213 DElncRNAs and 6809 DEmRNAs probesets were used to carry out WGCNA, by which 10 modules were generated. The most significant module (marked in black_M6) was selected for further analysis. Several critical biological processes were identified after PVL, such as IL-6 production, Wnt signaling pathway, and autophagy. Sixteen mRNAs (*Notch1*, *Grb2*, *IL-4*, *Cops4*, *Stxbp1*, *Khdrbs2*, *Hdac2*, *Gnb3*, *Gng10*, *Tlr2*, *Sod1*, *Gosr2*, *Rbbp5*, *Map3k3*, *Golga2*, and *Rev3l*) and ten lncRNAs (*BC092620*, *AB190508*, *EF076772*, *BC088302*, *BC158675*, *BC100646*, *BC089934*, *L20987*, *BC091187*, and *M23890*) were identified as hub genes based on gene significance value, module membership value, protein–protein interaction (PPI) and lncRNA-mRNA co-expression network. Finally, 3 mRNAs (*Notch1*, *Grb2* and *IL-4*) and 4 lncRNAs (*BC089934*, *EF076772*, *BC092620* and *BC088302*) were identified as promising targets for accelerating liver regeneration before extensive hepatectomy.

## Conclusions

Three mRNAs (*Notch1*, *Grb2*, and *IL-4*) and four lncRNAs (*BC089934*, *EF076772*, *BC092620*, and *BC088302*) may be promising targets for accelerating liver regeneration before or after extensive hepatectomy, as revealed by liver tissue microarray and WGCNA analysis in PVL animals and validated by liver tissue samples from PVE clinical patients.

## Supplementary Information

**Additional file 1:** **Supplementary Figure S1**. DEmRNAs and DElncRNAs probesets between control group and PVL group. (A) There were 3686 DEmRNAs probesets between control group and PVL day 1 (*p*-value < 0.05 and FC ≥ 2.0), 2965 DEmRNAs probesets between control group and PVL day 7, 3570 DEmRNAs probesets between control group and PVL day 14. (B) There were 2485 DElncRNAs probesets between control group and PVL day 1 (*p*-value < 0.05 and FC ≥ 2.0), 2391 DElncRNAs probesets between control group and PVL day 7, 2694 DElncRNAs probesets between control group and PVL day 14.

**Additional file 2:** **Supplementary Figure S2**. Hierarchical cluster analysis. (A) Hierarchical cluster analysis showed the expression variations of these hub mRNAs in lobe-pbs at different time points. (B) Hierarchical cluster analysis showed the expression variations of these hub lncRNAs in lobe-pbs at different time points.

**Additional file 3:** **Table S1**.

**Additional file 4:** **Table S2**.

**Additional file 5:** **Table S3**.

## Data Availability

All data generated or analysed during this study were included in this published article and its supplementary information files.

## Abbreviations

HCC: Hepatocellular carcinoma
FLR: Future liver remnant
PVL: Portal vein ligation
PVE: Portal vein embolization
IL-6: Interleukin-6 (IL-6)
TGF-α: Transforming growth factor-alpha
TGF-β: Transforming growth factor-beta
lobe-pbs: Hepatic lobes preserving portal blood supply
lncRNA: Long noncoding RNA
mRNA: Messenger RNA
ALPPS: Associated liver partition and portal vein ligation for staged hepatectomy
WGCNA: Weighted gene correlation network analysis
GO: Gene ontology
DEmRNAs: Differentially expressed mRNAs
DElncRNAs: Differentially expressed lncRNAs
SMMU: Second military medical university
EHBH: Eastern hepatobiliary surgery hospital
TOM: Topological overlap matrix
KEGG: Kyoto encyclopedia of genes and genomes
BP: Biological process
CC: Cellular component
MF: Molecular function
PPI-network: Protein–protein interaction network
GS: Gene significance
MM: Module membership
qRT-PCR: Quantitative real-time PCR
