# Supplementary figures and images for: Identification of crucial lncRNAs and mRNAs in liver regeneration after portal vein ligation through weighted gene correlation network analysis
Source: BMC Genomics. 2022 Sep 21;23:665. doi: 10.1186/s12864-022-08891-0 (PMC9490934; doi:10.1186/s12864-022-08891-0)

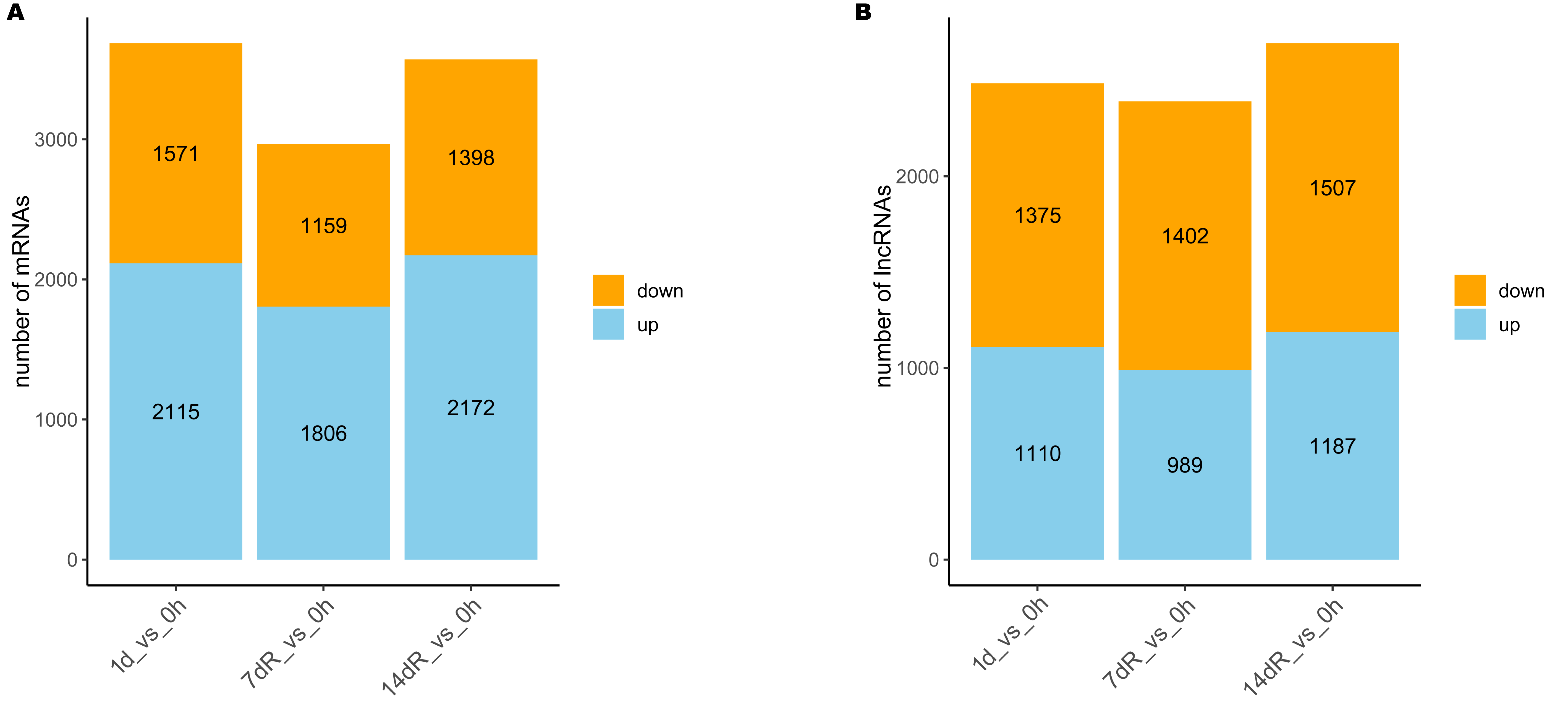

Supplement: Supplementary file 1 — Additional file 1: Supplementary Figure S1. DEmRNAs and DElncRNAs probesets between control group and PVL group. (A) There were 3686 DEmRNAs probesets between control group and PVL day 1 (p-value < 0.05 and FC ≥ 2.0), 2965 DEmRNAs probesets between control group and PVL day 7, 3570 DEmRNAs probesets between control group and PVL day 14. (B) There were 2485 DElncRNAs probesets between control group and PVL day 1 (p-value < 0.05 and FC ≥ 2.0), 2391 DElncRNAs probesets between control group and PVL day 7, 2694 DElncRNAs probesets between control group and PVL day 14. [file 12864_2022_8891_MOESM1_ESM.tif]

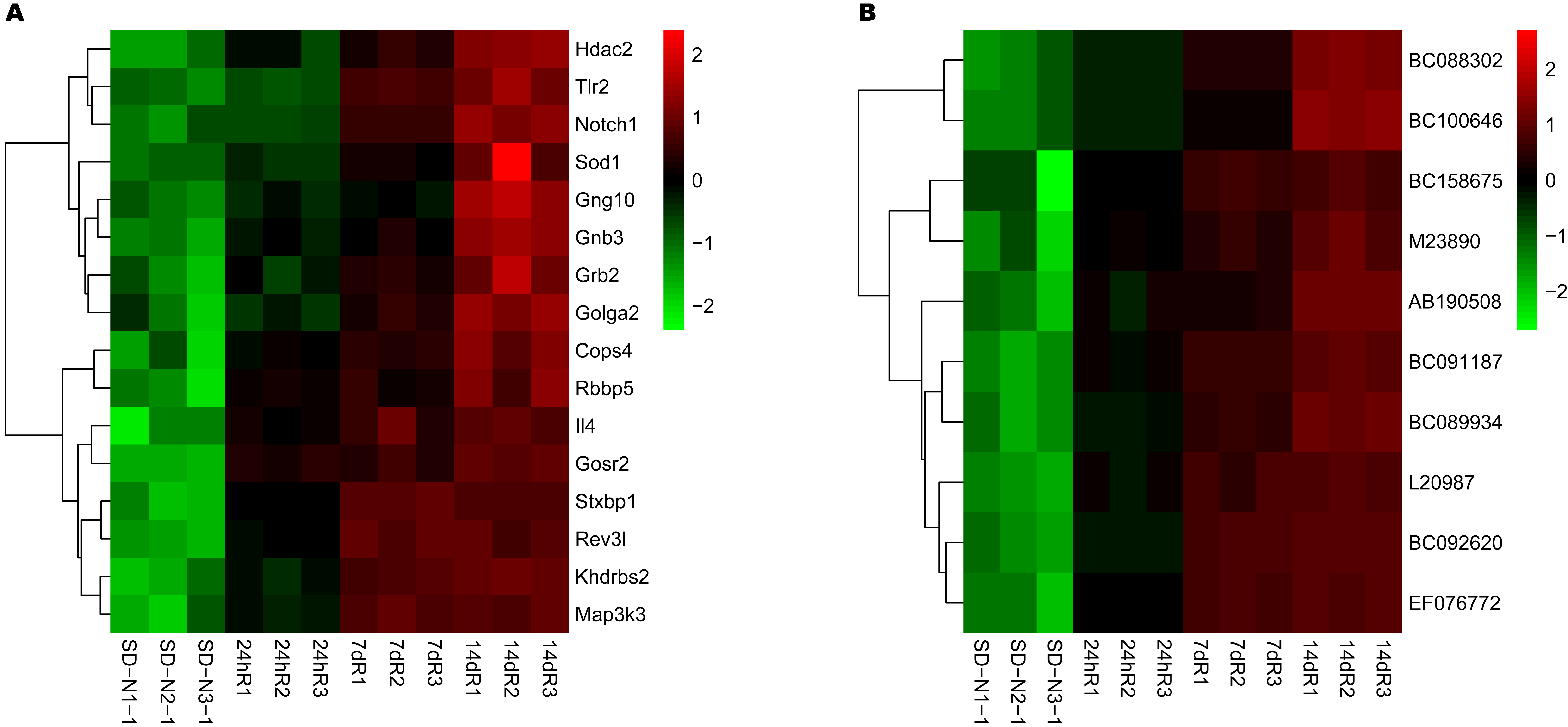

Supplement: Supplementary file 2 — Additional file 2: Supplementary Figure S2. Hierarchical cluster analysis. (A) Hierarchical cluster analysis showed the expression variations of these hub mRNAs in lobe-pbs at different time points. (B) Hierarchical cluster analysis showed the expression variations of these hub lncRNAs in lobe-pbs at different time points. [file 12864_2022_8891_MOESM2_ESM.tif]
